# Successful Resection of a Re-Occurred Pulmonary Myosarcoma in a Patient with Turner Syndrome Mosaic

**DOI:** 10.1080/1357714021000066395

**Published:** 2002-12

**Authors:** Volker F. H. Brauer, Frank Reichenberger, Anke Müller, Matthias Steinert, Ursula G. Froster, Hubert R. W. Wirtz, Joachim Schauer

**Affiliations:** 1 Division of Pneumology Department of Medicine University Hospital Leipzig Leipzig Germany; 2 Institute of Pathology University Hospital Leipzig Leipzig Germany; 3 Division of Thoracic Surgery Department of Surgery University Hospital Leipzig Leipzig Germany; 4 Institute of Human Genetics University Hospital Leipzig Leipzig Germany; 5 Department of Internal Medicine III University Hospital Leipzig Philipp-Rosenthal-Str. 27 Leipzig 04103 Germany

## Abstract

We describe a patient who underwent thoracic radiation therapy for biopsy-proven pulmonary spindle cell sarcoma in the left lower lobe, 15 months after birth. At the age of 37 she developed shoulder pain, fatigue, and progressive exertion dyspnoea. Chest X-ray revealed a pulmonary mass in the left lower lobe due to a cytology-proven malignant tumour.The patient underwent left pneumonectomy. Histology revealed a myosarcoma of the lung, similar to the previous sarcoma. Furthermore, the patient was diagnosed to have Turner syndrome mosaic and chromosomal analysis revealed a translocation t(1;13) in 3/50 metaphases. However a germline mutation of the p53 tumour suppressor gene was excluded. After 2 years of follow-up the patient is stable and there are no signs of recurrence of the tumour.We conclude a re-occurrence of this very rare malignant disorder of the lung after a 36-year interval in a patient with Turner syndrome mosaic. Following initial curative radiation therapy, with a remission over 36 years, lung resection was now successfully performed.


*Sarcoma* (2002) **6**, 141–143

Taylor & Francis healthsciences

**CASE REPORT**

### Successful resection of a re-occurred pulmonary myosarcoma in a patient with Turner syndrome mosaic

VOLKER F.H. BRAUER[1], FRANK REICHENBERGER[1], ANKE MÜLLER[2], MATTHIAS STEINERT[3], URSULA G. FROSTER[4], HUBERT R.W. WIRTZ[1] & JOACHIM SCHAUER[1]

*[1]Division of Pneumology, Department of Medicine, [2]Institute of Pathology, [3]Division of Thoracic Surgery, Department of Surgery and [4]Institute of Human Genetics, University Hospital Leipzig, Leipzig, Germany*

**Abstract**

We describe a patient who underwent thoracic radiation therapy for biopsy-proven pulmonary spindle cell sarcoma in the left lower lobe, 15 months after birth. At the age of 37 she developed shoulder pain, fatigue, and progressive exertion dyspnoea. Chest X-ray revealed a pulmonary mass in the left lower lobe due to a cytology-proven malignant tumour. The patient underwent left pneumonectomy. Histology revealed a myosarcoma of the lung, similar to the previous sarcoma. Furthermore, the patient was diagnosed to have Turner syndrome mosaic and chromosomal analysis revealed a translocation t(1;13) in 3/50 metaphases. However a germline mutation of the p53 tumour suppressor gene was excluded. After 2 years of follow-up the patient is stable and there are no signs of recurrence of the tumour. We conclude a re-occurrence of this very rare malignant disorder of the lung after a 36-year interval in a patient with Turner syndrome mosaic. Following initial curative radiation therapy, with a remission over 36 years, lung resection was now successfully performed.

**Key words:** *pulmonary myosarcoma, pulmonary hypertension, Turner syndrome*

### Case report

A 37-year-old female was admitted with a 3-month history of fatigue, progressive exertion dyspnoea and left shoulder pain. Chest X-ray revealed a large pulmonary process in the left hemi-thorax. One month before she underwent cardiac catheterisation, and severe pulmonary arterial hypertension was diagnosed with a mean pulmonary artery pressure of 75 mmHg. There were no signs of coronary or valvular heart disease.

At the age of 15 months she had a biopsy-proven spindle cell soft tissue sarcoma in the posterior mediastinum. Because of chest wall invasion a resection could not be performed, and she received percutaneous thoracic radiation with a total dose of 40 Gy. In 1998, she underwent hysterectomy for a benign myoma, and an uterus duplex and a vagina subsepta were found. In addition, she was diagnosed for primary amenorrhoea.

On examination the patients height was 155 cm. She had a hypoplastic left hemi-thorax and mammary gland, most likely as a result of the radiation therapy in childhood. Auscultation revealed dullness and rales over the base of the left lung. A 4/6 systolic cardiac murmur on the left parasternal was present. Laboratory examinations were within normal limits.

Pulmonary function tests showed a severe restrictive pattern (TLC 2.6 l; [56% predicted]), but capillary blood gases were normal ($pCO_2$ 5.8 kPa, $pO_2$ 10.3 kPa). Diffusion capacity was impaired, with a transfer factor for carbon monoxide of 53% predicted, consistent with restrictive lung disease. Pulmonary scintigraphy showed a highly reduced perfusion of the left lung with a distribution of 8% on the left and 92% on the right lung, but no signs of pulmonary embolism.

Thoracic MRI revealed a $10 \times 7 \times 5$cm lesion extending to the pericardium, the diaphragm and the thoracic wall without signs of infiltration (Fig. 1).

There were no focal lesions detected on abdominal ultrasound, bronchoscopy, skeletal scintigraphy and liver MRI.

Ultrasound-guided transthoracic biopsy of the tumour revealed a malignant sarcoma.

The patient underwent left pneumonectomy with partial resection of the diaphragm and the pericardium due to tumour adhesion. Intraoperatively, a patent ductus Botalli (PDA) was found and subsequently occluded.

Histology revealed a pleomorphic myogenic sarcoma of the lung neither infiltrating the pleura nor metastasising into the hilar or mediastinal lymph

Correspondence to: Volker F.H. Brauer, Department of Internal Medicine III, University Hospital Leipzig, Philipp-Rosenthal-Str. 27, 04103 Leipzig, Germany. Tel.: +49-341-97-13116; Fax: +49-341-97-13119; E-mail: vbrauer@web.de

ISSN 1357–714X print/ISSN 1369–1643 online/02/0200141–03 © Taylor & Francis Ltd
DOI: 10.1080/1357714021000066395

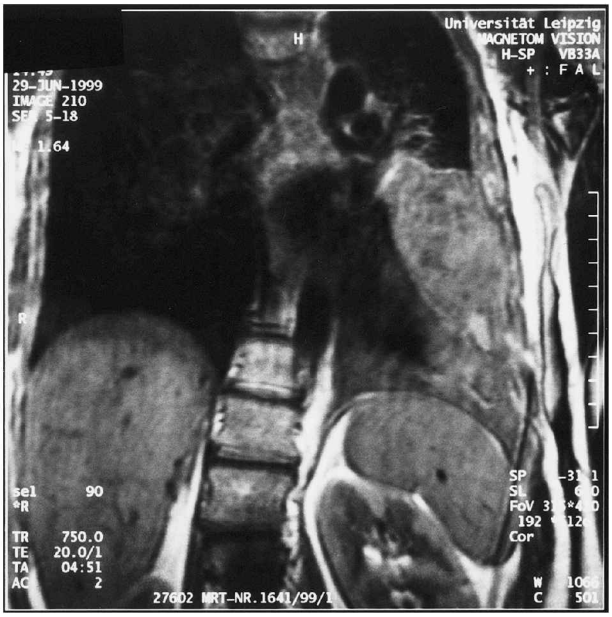

**Fig. 1.** *MRI presents an approximately 10 × 7 × 5cm lesion extending to the pericardium, the diaphragm and the thoracic wall, without signs of infiltration of the tumour. Furthermore, there is a hypoplasia of the left hemithorax and a dilatation of the right heart.*

nodes (stage I, Table 1; Fig. 2). Immunohistology was positive for vimentin, desmin, α-sarcomere actin and anti-muscle actin and negative for α-smooth muscle actin. The proliferation rate was nearly 60%.

A germline p53 mutation was excluded by automated sequencing of exons 4–10 of the TP 53 gene (ABI 377, Perkin-Elmer®). Chromosomal analysis of peripheral blood revealed an abnormal karyotype with a mosaic 45,X/46,XX,t(1;13)(p36;q14)/46,XX, consistent with a cytogenetic variant of Turner syndrome.

The further hospital stay was without complications. One month postoperatively, pulmonary artery pressure had decreased to 60 mmHg by echocardiography. At 2-year follow-up, the patient remained stable without radiological signs of recurrence of the tumour. Pulmonary artery pressure further decreased to 35 mmHg by echocardiography.

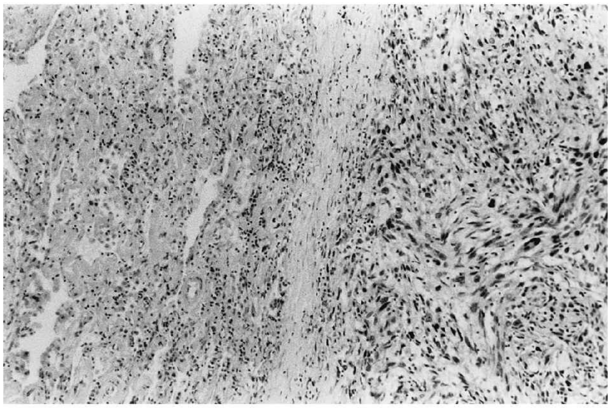

**Fig. 2.** *Histology reveals an undifferentiated spindle cell like sarcoma with multiple mitosis on the left side. On the right side there is normal lung tissue with terminal bronchioles.*

### Discussion

Rhabdomyosarcoma is a typical extracranial solid malignant tumour presenting during childhood. Every year, approximately 250 cases are diagnosed in the United States. In Germany, 60–70 cases are found annually with an incidence of 0.5%.[2,3] An intrathoracic manifestation of RMS is very rare.[4]

Surgical resection is the only curative therapy. Radiation therapy is indicated in microscopic or residual disease, whereas chemotherapy is suggested for cytoreduction and eradication of micrometastases. Multimodality treatment protocols, used in clinical studies, enable disease-free survival up to 70%, in cases with incomplete resection.[2,5] At recurrence of the sarcoma, 5-year survival is 40% for stage I versus 3% for any higher stage.[6]

In our patient, radiation therapy of inoperable sarcoma during childhood induced complete remission and cure from the malignant disease for 36 years.[7]

At the reoccurrence of the myosarcoma the patient presented with localized disease. The tumour was completely resected, and there was no indication for further anticancer therapy. Left pneumonectomy was feasible despite the impaired pulmonary function, because the left lung contributed only 8% to pulmonary blood flow and oxygenation.[8] However, there

**Table 1.** *Clinical group stage system for rhabdomyosarcoma (1)*

| Clinical group | Extent of disease/surgical result |
|---|---|
| I | A. Localized tumour, confined to site of origin, completely resected. |
| | B. Localized tumour, infiltrating beyond site of origin, completely resected. |
| II | A. Localized tumour, gross total resection, but with microscopic residual disease. |
| | B. Locally extensive tumour (spread to regional lymph nodes), completely resected. |
| | C. Locally extensive tumour (spread to regional lymph nodes), gross total resection, but microscopic residual disease. |
| III | A. Localized or locally extensive tumour, gross residual disease after biopsy only. |
| | B. Localized or locally extensive tumour, gross residual disease after major resection (50% debulking). |
| IV | A. Any size primary tumour, with or without regional lymph node involvement, with distant metastases, irrespective of surgical approach to primary tumour. |

was a considerable perioperative risk because of the severe pulmonary arterial hypertension. Surgical closure of the PDA was feasible after exclusion of an Eisenmenger syndrome resulting in a postoperative reduction of the pulmonary artery pressure.

We regard the present tumour as a re-occurrence of a similar tumour type in a high-risk constellation rather than a relapse after 36 years. Myosarcomas have a high local recurrence rate, and both the recent and the childhood tumour showed close histological similarities. Furthermore, chromosomal analysis was consistent with a variant of Turner syndrome. This condition is associated with a higher rate of nongonadal malignancies.[9,10] Our Turner syndrome-affected patient had the following clinical anomalies: short in stature (155 cm), primary infertility, heart defect (persistent ductus Botalli), hypoplastic left hemi-thorax and mammary gland. There were no signs like epicanthal folds, low nuchal hair line, shield-like chest, webbed neck, high arched palate, renal anomalies, pigmented nevi or lymphedema.

The previous radiation therapy could also be regarded as a risk factor for developing a secondary malignancy.[11,12] However, a p53 germline mutation, often found in early childhood mesenchymal tumours, was not present in our patient.[13,14]

### Conclusion

We report the successful lung resection of a re-occurring pulmonary myosarcoma in a patient with Turner syndrome. A previous radiation therapy resulted in a complete remission for 36 years. The condition of an earlier myosarcoma, previous radiation therapy, and Turner syndrome has to be regarded as a high risk constellation for a secondary malignancy.

### References

1 Andrassy RJ, Wiener ES, Raney RB, Lawrence W, Lobe TE, Corpron CA, Maurer HM, Thoracic sarcomas in children. *Ann Surg* 1998; 227: 170–3.

2 Dagher R, Helman L. Rhabdomyosarcoma: an overview. *The Oncologist* 1999; 4: 34–44.

3 Pedrick TJ, Donaldson SS, Cox RS. Rhabdomyosarcoma: the Stanford experience using a TNM staging system. *J Clin Oncol* 1986; 4: 370–8.

4 Badreddine J, Ledoux JP, Marcade E, Caulet-Maugendre S. Primary pulmonary rhabdomyosarcoma: a case report. *Ann Pathol* 2000; 20 (1): 66–8.

5 Tierney JF, Mosseri V, Stewart LA, Souhami RL, Parmar MK. Adjuvant chemotherapy for soft-tissue sarcoma: review and meta-analysis of the published results of randomised clinical trials. *Br J Cancer* 1995; 72: 469–75.

6 Pappo AS, Anderson JR, Crist, WM, *et al.* Survival after relapse in children and adolescents with rhabdomyosarcoma: a report from the Intergroup rhabdomyosarcoma study group. *J Clin Oncol* 1999; 17: 3487–93.

7 Persic M, Roberts JT, Alveolar rhabdomyosarcoma metastatic to the breast: long-term survivor. *J Clin Oncol* 1999; 11: 417–8.

8 Bolliger CT, Perruchoud AP. Functional evaluation of the lung resection candidate. *Eur Respir J* 1998; 11: 198–212.

9 Blatt J, Olsban AF, Lee PA, Ross, JL. Neuroblastoma and related tumours in Turner's syndrome. *J Pediatr* 1997; 131: 666–70.

10 Wertelecki W, Fraumeni JF Jr, Mulvihill JJ. Nongonadal neoplasia in Turner's syndrome. *Cancer* 1970; 26: 485–8.

11 Heyn R, Haeberlen V, Newton WA, Ragab AH, Raney RB, Tefft M, Wharam M, Ensign LG, Maurer HM. Second malignant neoplasms in children treated for rhabdomyosarcoma. *J Clin Oncol* 1993; 11, 262–70.

12. Raney RB, Asmar L, Vassilopoulou-Sellin R, Klein MJ, Donaldson SS, Green J, Heyn R, Wharam M, Glicksman AS, Gehan EA, Anderson J, Maurer HM. Late complications of therapy in 213 children with localized, nonorbital soft-tissue sarcoma of the head and neck: a descriptive report from the Intergroup Rhabdomyosarcoma Studies (IRS)-II and -III. IRS Group of the Children's Cancer Group and the Pediatric Oncology Group. *Med Pediatr Oncol* 1999; 33: 362–71.

13. Densmore TL, Langer JC, Molleston JP, Dehner LP, Coffin CM, Lauren V. Colorectal adenocarcinoma as a second malignant neoplasm following Wilms' tumour and rhabdomyosarcoma. *Med Pediatr Oncol* 1996; 6: 556–60.

14. El Badry OM, Minniti C, Kohn EC, *et al.* Insulin like growth factor II acts as an autocrine growth and motility factor in human rhabdomyosarcoma tomours. *Cell Growth Differ* 1990; 1: 325–31.
